# Comparison of MMP-2, MMP-9, COX-2, and PGP Expression in Feline Injection-Site and Feline Noninjection-Site Sarcomas—Pilot Study

**DOI:** 10.3390/ani14142110

**Published:** 2024-07-19

**Authors:** Agata Wojtkowska, Anna Małek, Sławomir Giziński, Rafał Sapierzyński, Anna Rodo, Justyna Sokołowska, Katarzyna A. Zabielska-Koczywąs, Anna Wojtalewicz, Magdalena Walewska, Ewa Kautz, Magdalena Ostrzeszewicz, Roman Lechowski

**Affiliations:** 1 Department of Small Animal Diseases with Clinic, Institute of Veterinary Medicine, Warsaw University of Life Sciences, 02-787 Warsaw, Poland; anna_malek@sggw.edu.pl (A.M.); katarzyna_zabielska@sggw.edu.pl (K.A.Z.-K.); anna_wojtalewicz@sggw.edu.pl (A.W.); magdalena_walewska@sggw.edu.pl (M.W.); magdalena_ostrzeszewicz@sggw.edu.pl (M.O.); roman_lechowski@sggw.edu.pl (R.L.); 2 Department of Large Animal Diseases with Clinic, Institute of Veterinary Medicine, Warsaw University of Life Science, 02-787 Warsaw, Poland; slawomir_gizinski@sggw.edu.pl (S.G.); ewa_kautz@sgww.edu.pl (E.K.); 3 Department of Pathology and Veterinary Diagnostics, Institute of Veterinary Medicine, Warsaw University of Life Sciences, 02-787 Warsaw, Poland; rafal_sapierzynski@sggw.edu.pl (R.S.); anna_rodo@sggw.edu.pl (A.R.); 4 Department of Morphological Sciences, Institute of Veterinary Medicine, Warsaw University of Life Sciences, 02-787 Warsaw, Poland; justyna_sokolowska@sggw.edu.pl

**Keywords:** cyclooxygenase-2, feline injection-site sarcoma, fibrosarcoma, P-glycoprotein, immunohistochemistry, matrix metalloprotease

## Abstract

**Simple Summary:**

Simple Summary: The aim of our research was to assess the expression of the selected proteins involved with inflammation and carcinogenesis, in order to expand knowledge of FISS and non-FISS. Matrix metalloproteinase-2, matrix metalloproteinase-9, cyclooxygenase-2, and P-glycoprotein were evaluated with the immunohistochemistry method. Our results showed that the expressions of COX-2, MMP-9, and PGP were significantly higher in FISS than in non-FISS

**Abstract:**

Feline injection-site sarcomas (FISSs) are aggressive neoplasms that have been associated mostly with vaccination. Feline noninjection-site sarcomas (non-FISSs) are less frequently observed in cats and may arise in any anatomic site. This study aimed to determine the differences in the expression of the selected proteins (matrix metalloproteinase-2 (MMP-2), matrix metalloproteinase-9 (MMP-9), cyclooxygenase-2 (COX-2), and P-glycoprotein (PGP)) and their correlation with the mitotic count in FISS and non-FISS, in order to characterize their immunohistochemical features. A preliminary study of eleven samples of FISS and eight samples of non-FISS was performed using immunohistochemistry. Among all the tested sarcomas, 80.4% of the tumors were positive for COX-2, 90.2% were positive for MMP-9, and 100% were positive for PGP. The results showed that the expressions of COX-2, MMP-9, and PGP were significantly higher in FISS than in non-FISS (COX-2—*p* ≤ 0.001; MMP-9—*p* ≤ 0.05; and PGP—*p* ≤ 0.05). A Spearman rank correlation analysis showed a moderate negative correlation between the expression of COX-2 and MMP-9 in FISS (r = −0.52). A strong negative correlation between COX-2 and PGP (r = −0.81), a moderate positive correlation between MMP-2 and MMP-9 (r = +0.69), and a moderate negative correlation between MMP-2 and PGP (r = −0.44) were observed in non-FISS. In summary, our study presents the immunohistochemical profile of the proteins involved with inflammation and carcinogenesis in FISS and non-FISS, which can contribute to expanding the knowledge of tumor biology.

## 1. Introduction

Feline soft tissue sarcoma (STS) is one of the most common cutaneous neoplasms in cats [1,2]. Feline noninjection-site sarcoma (non-FISS), which may arise in any anatomical site, is characterized by a locally expansive growth pattern and has a low-to-moderate local recurrence rate following surgical excision [3]. Feline injection-site sarcoma (FISS) originates from the mesenchymal tissue and represents one of the most serious adverse effects after the injections of vaccines, nonsteroidal anti-inflammatory drugs, antibiotics, and hormones, as well as the recent introduction of surgical sponges, non-absorbable sutures, and microchips [4,5,6,7,8,9].

The etiopathogenesis of FISS is still unknown; however, the influence of locally chronic inflammatory factors and genetic predisposition are suspected [1,7,10]. FISS is a disease with a tendency to relapse after surgical removal in almost 60% of cases [11]. Tumors require aggressive surgical and adjuvant therapy: chemotherapy, immunotherapy, and/or radiotherapy [12]. The precise molecular cause of FISS needs elucidation, as it may be crucial to improving the current prognosis and therapy. 

Cyclooxygenase, a cyclic prostaglandin peroxide synthase, promotes the conversion of arachidonic acid into a variety of prostaglandins [13]. Prostaglandins are involved in the development of neoplasms [14]. Cyclooxygenase-2 (COX-2) is primarily expressed during cell growth, differentiation, inflammation, and carcinogenesis [15]. It has been shown useful as a prognostic factor in many histological types of tumors in humans [16].

Matrix metalloproteinases (MMPs) are a family of zinc metalloendopeptidases responsible for the turnover of matrix components. The physiological role of MMPs includes neurite growth, cell migration, and angiogenesis [17,18,19]. MMPs are involved in pathological processes such as tumor growth and migration, fibrosis, and arthritis. Recently, in both human and veterinary oncology, metalloproteinase activity has been investigated as a possible independent prognostic marker for tumors [19,20,21,22,23,24]. MMP-2 and MMP-9 high expression is related to the higher metastatic potential of several malignancies in humans, including breast, colon, and gastric carcinomas [25,26,27,28]. Only a few studies have evaluated MMP-2 and MMP-9 expression levels in FISS and non-FISS [9,29].

Transportation by ATP-dependent efflux pumps such as P-glycoprotein (PGP), encoded by genes associated with multidrug resistance, is a well-known mechanism that allows cells to maintain substrate homeostasis but also to evade drug therapy [30,31]. PGP plays a significant role in multidrug resistance in both human and animal tumors [31,32]. However, there is a limited number of studies assessing PGP expression in cats. PGP is expressed in 93.4% of feline mammary carcinomas, and it is positively associated with the tumor grade [33]. However, Brenn et al. (2008) [34] reported no correlation between PGP expression and the disease-free interval or overall survival associated with feline lymphoma. Positive PGP expression was also found in feline primary pulmonary carcinoma but with no relation to histopathological characteristics [35]. To the best of our knowledge, no study has investigated PGP expression in FISS and non-FISS.

Therefore, this study aimed to immunohistochemically characterize FISS and non-FISS in cats, to create a marker profile potentially associated with carcinogenesis, metastasis, and drug resistance. The immunohistochemical expression of COX-2, PGP, MMP-2, and MMP-9 and their differences between FISS and non-FISS were evaluated.

## 2. Materials and Methods

### 2.1. Sample Collection

This study included 19 cutaneous tumors collected from the archive of our university in years 2008–2015. Sections of skin and subcutaneous tissue tumors were classified as fibrosarcomas during the routine evaluation of preparations stained with Mayer’s hematoxylin and eosin (HE) by the Division of Animal Pathomorphology, Faculty of Veterinary Medicine, Warsaw University of Life Sciences. The study group comprised 11 samples of FISS and 8 samples of non-FISS. Each tumor was examined in a blind manner by two specialized veterinary pathologists. To be included in the study, tumors had to meet specific criteria: to have arisen from the tissue at sites of previous vaccination in one of several specific anatomical locations (the interscapular region, the lateral abdominal, or thoracic wall or the lumbar area) and displaying the presence of characteristic histological features such as lymphocytes clusters, tissue infiltration by tumor cells, necrosis in the tumor parenchyma, scarring areas in the surrounding tissues, inflammatory infiltration in the surrounding tissues of the tumor mass, adjuvants in macrophages, a moderate or high mitotic index, and multinuclear giant cells [36,37]. Non-FISSs were classified based on the following: no information about previous injections at the tumor site as per the clinical history; a tumor arisen in a location atypical for vaccination (mammary gland, armpit area, the inside of the thigh, facial area, ears); and no characteristic features of FISS during the histological examination.

### 2.2. Mitotic Count (MC)

The mitotic count (MC) was evaluated in a blind manner. The MC was calculated based on the number of mitoses per 10 contiguous high-power fields (HPFs)/2.37 mm^2^ and presented as a score (1 = 1–9 mitoses, 2 = 10–19 mitoses, 3 = ≥20 mitoses). The calculation was performed avoiding the areas of necrosis or severe inflammation (40× objective) [38].

### 2.3. Immunohistochemical Staining

The sections for immunohistochemistry were cut into 3 μm sections, mounted on hydrophilic slides (Hydrophilic Plus Microscope Slides; Bio SB, Santa Barbara, CA, USA), and dried at 42 °C for 24 h. After dewaxing in xylene and rehydration in ethanol, the slides were heated in a microwave (for 7 and then 5 min) in 0.02 M citrate buffer (pH 6.0) for antigen retrieval. After cooling, the sections were incubated in 3% perhydrol solution at room temperature for 15 min to block endogenous peroxidase activity. Nonspecific binding was blocked by incubation with 5% bovine serum albumin for 30 min (Sigma Aldrich, Schnelldorf, Germany). An immunohistochemical examination of each tumor was performed using primary antibodies (diluted in 1% bovine serum) described previously in feline tumors: COX-2 [39,40] (monoclonal mouse anti-human, clone CX-294; dilution 1:50, incubation time: 1 h in a humid chamber at room temperature; Dako, Glostrup, Denmark), MMP-2, MMP-9 [21] (mouse monoclonal antibodies AB3158 clone CA-4001/CA719E3C; AB58803 clone 56-2A4; dilution 1:100; incubation time: 1 h in a humid chamber at room temperature; Abcam, Cambridge, UK), and PGP [41,42] (clone C494; dilution 1:100; incubation time: 1 h in a humid chamber at room temperature; Covance, Dedham, MA, USA). A visualization system based on the method with 3,3-diaminobenzidine (DAB) as a substrate (EnVision Detection System, Peroxidase/DAB+, Rabbit/Mouse, Dako, Glostrup, Denmark) was used. The sections were counterstained with Ehrlich’s hematoxylin for 10 min. Then, they were dehydrated in a series of increasing concentrations of alcohol, cleared in xylene, and mounted using DPX medium (Gurr^®^; Sigma Aldrich, Schnelldorf, Germany). Positive control (COX-2—inflamed skin tissue, MMP-2 and MMP-9—feline osteosarcoma, PGP—unchanged cat liver) and negative control (a mixture of tris-buffered saline and polysorbate 20 (TBST) (Dako, Glostrup, Denmark) was used instead primary antibodies) slides were processed together with the evaluated slides. Brown staining in the cytoplasm was considered as a positive reaction. 

### 2.4. Immunohistochemical Evaluation

The immunostaining was blindly evaluated by two operators. An immunohistochemical analysis was performed to assess MMP-2, MMP-9, COX-2, and PGP expressions of at least 10 HPFs at a 400 magnification using an Axio Imager A2 microscope (ZEISS, Oberkochen, Germany).

### 2.5. COX-2 Evaluation

COX-2 immunoreactivity was defined by a scoring system based on the percentage of positive cells and the staining intensity. The score of positively stained cells was evaluated using the following: 0—negative; 1—less than 10% of cells stained positive; 2—10% to 30% of cells stained positive; 3—31% to 60% of cells stained positive; and 4—more than 60% of cells stained positive. The intensity score was evaluated using the following: 0—negative; 1—weak staining; 2—moderately intense staining; and 3—intense staining [13,42]. The final result was presented as the multiplication of the intensity score and the percentage score:

immunoreactivity=intensity score×percentage score 

### 2.6. MMP-2 and MMP-9 Evaluation

The expression of MMP-2 and MMP-9 was assessed using the semiquantitative scale proposed by Aresu et al. [43], which included the intensity of the immunostaining score (0—no labeling detected; 1—weak-to-moderate labeling; 2—moderate-to-strong labeling and 3—strong labeling) and the percentage of positive cells. The multiplication of the intensity and percentage of positive cells was considered the final result [44].

immunoreactivity=intensity score×percentage of positive cells [%]

### 2.7. PGP Evaluation

The PGP immunoreactivity was defined by quantifying the percentage score of positively stained cells and the staining intensity score in the entire section, using the immunoreactivity scoring system [33]. The immunoreactivity was defined as follows:

immunoreactivity=intensity score×percentage score

The staining intensity score was classified as follows: 0 = negative; 1 = weak; 2 = moderate; and 3 = strong. The score of positively stained cells was defined as follows: 0 = no signal; 1 = up to 10%; 2 = 10% to 50%; and 3 = 50% or more. Samples with a score of 2 or 3 were considered positive. 

### 2.8. Statistical Analysis

The data were analyzed using GraphPad Prism 8.0 (San Diego, CA, USA) and the Mann–Whitney U test to assess the differences in immunoreactivity for MMP-2, MMP-9, COX-2, and PGP between the non-FISS and FISS groups. Significance was considered when *p* < 0.05, whereas high significance was considered when *p* < 0.01 and *p* < 0.001.

The association between variables for FISS and non-FISS was assessed using the Spearman correlation matrix. The correlation was classified as follows: none, less than 0.10; weak, 0.10 to 0.39; moderate, 0.40 to 0.69; strong, 0.70 to 0.99; and perfect, 1.0 [45,46].

## 3. Results

### 3.1. Characteristics of Evaluated Samples

The results of the FISS and non-FISS tumors assessment are described in Table 1, which provides a summary of the clinicopathological, histopathological, and immunohistochemical features of the tumors. Supplementary Material S1 includes comprehensive data on the staining intensity and the percentage of positively stained cells for every each tumor.

### 3.2. Mitotic Count (MC)

In 4 of 11 FISSs (36.36%), we observed >20 mitoses (MC = 3), and 7 out of all FISSs (57.14%) had between 10 and 19 mitoses (MC = 2) in 2.37 mm^2^. For non-FISSs, two of eight (25%) had ≥20 mitoses (MC = 3), and six of eight (75%) presented from 10 to 19 mitoses (MC = 2) in 2.37 mm^2^ (Table 2).

### 3.3. Evaluation of COX-2 Expression

**Figure 1:**
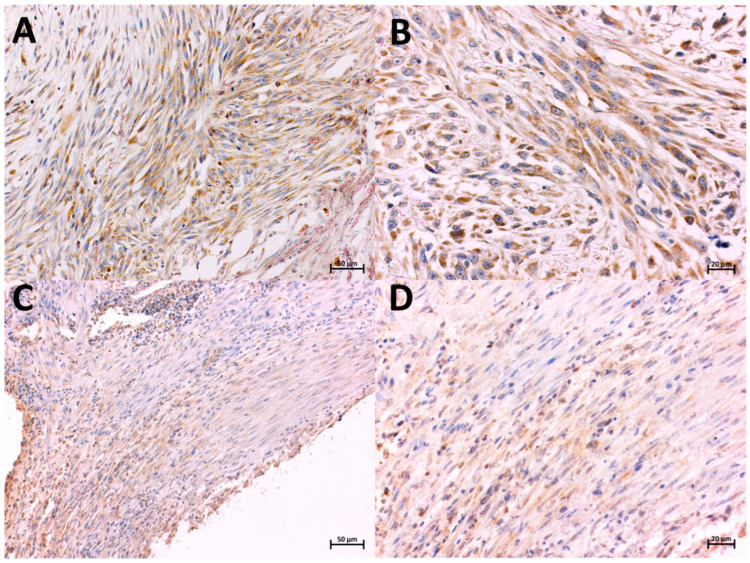
Localization of COX-2-expressing cells in FISS (**A**,**B**) and non-FISS (**C**,**D**), immunohistochemically stained. Brown color indicates positive staining. Positive immunostaining for COX-2 in FISS, intensity 3, 90% of positive cells, magnification 20× and 40×, respectively (**A**,**B**). Positive immunostaining for COX-2 in non-FISS, intensity 1, 60% of positive cells, magnification 20× and 40×, respectively (**C**,**D**).

**Figure 2:**
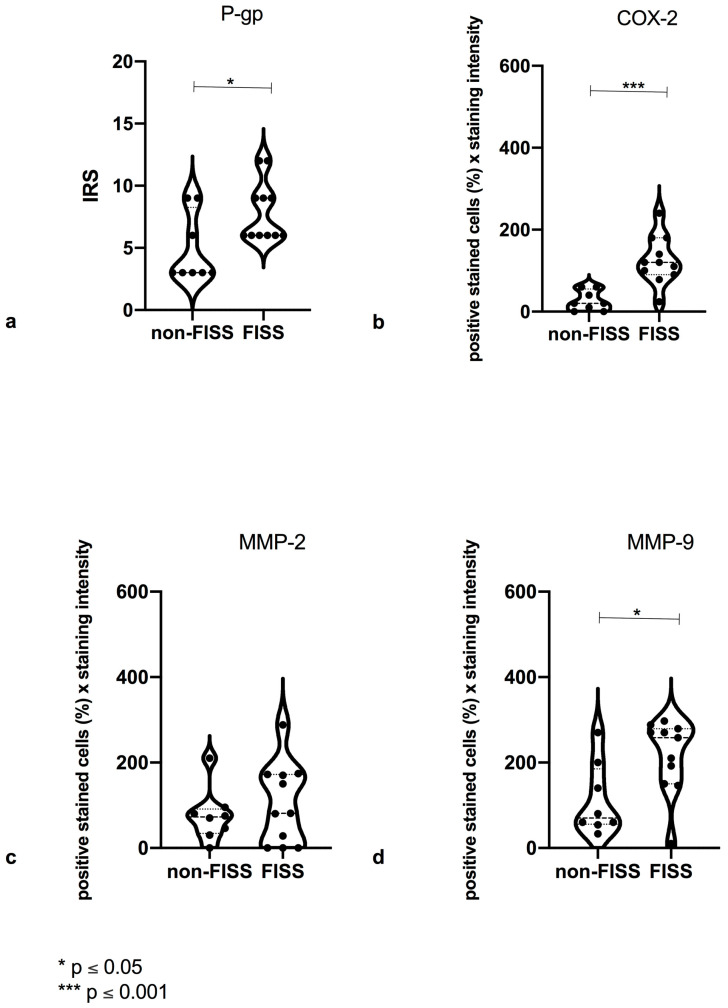
Statistical analysis of P-glycoprotein (PGP) (**a**), cyclooxygenase 2 (COX-2) (**b**), matrix metalloproteinase-2 (MMP-2) (**c**), and matrix metalloproteinase-9 (MMP-9) (**d**) expressions in evaluated groups. (**a**) Significant (*) (*p* ≤ 0.05) difference in PGP expression with feline injection-site sarcoma (FISS) and with feline noninjection-site sarcoma (non-FISS). (**b**) Significantly higher (***) (*p* ≤ 0.001) expression of COX-2 with FISS and non-FISS. (**c**) No significant difference in MMP-2 expression with FISS and non-FISS. (**d**) Significantly (*) (*p* ≤ 0.05) higher expression of MMP-9 with FISS than with non-FISS.

The expression of COX-2 was predominantly observed in the cytoplasm of mononucleated spindle cells (Figure 1A,B). Among all sarcomas (FISS and non-FISS), 80.4% were positive for COX-2. The mean (±standard deviation SD]) COX-2 expression values were 26.25 (SD, ±24.46) and 125.6 (SD, ±58.34) for non-FISS and FISS, respectively, corresponding to a highly significant (*p* = 0.001) difference between them (Figure 2).

### 3.4. Evaluation of MMP-2 Expression

**Figure 3:**
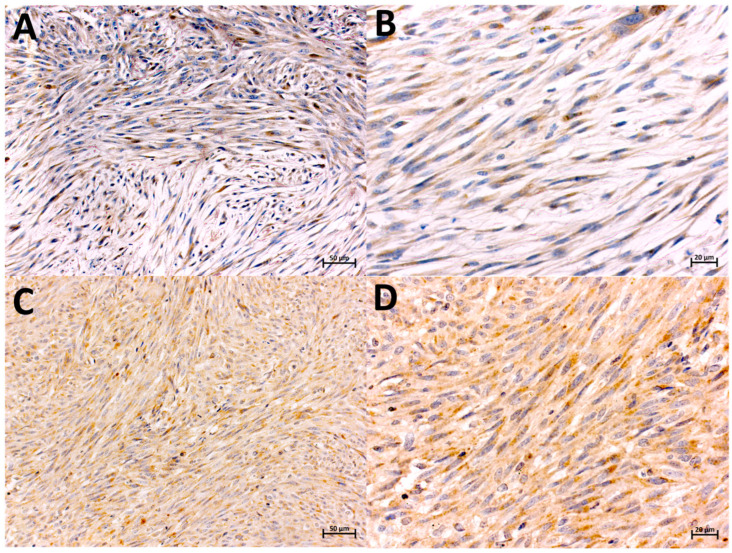
Localization of MMP-2-expressing cells in FISS (**A**,**B**) and non-FISS (**C**,**D**), immunohistochemically stained. Brown color indicates positive staining. Positive immunostaining for MMP-2 in FISS, intensity 2, 87% of positive cells, magnification 20× and 40×, respectively (**A**,**B**). Positive immunostaining for MMP-2 in non-FISS, intensity 1, 90% of positive cells, magnification 20× and 40×, respectively (**C**,**D**).

MMP-2 expression was predominantly observed in the cytoplasm of mononucleated spindle cells (Figure 3A,B). Among all sarcomas (FISS and non-FISS), 17.64% of the tumors were negative and 82.36% were positive for MMP-2. The mean MMP-2 expression values were 75.75 (SD, ±62.29) and 103.9 (SD, ±94.44) for non-FISS and FISS, respectively. Positive staining was observed in the endothelial cells of the peritumoral small vessels, particularly in perivascular lymphoid aggregates. The statistical analysis revealed no significant difference in the MMP-2 expression of the FISS and non-FISS groups (Figure 2).

### 3.5. Evaluation of MMP-9 Expression

**Figure 4:**
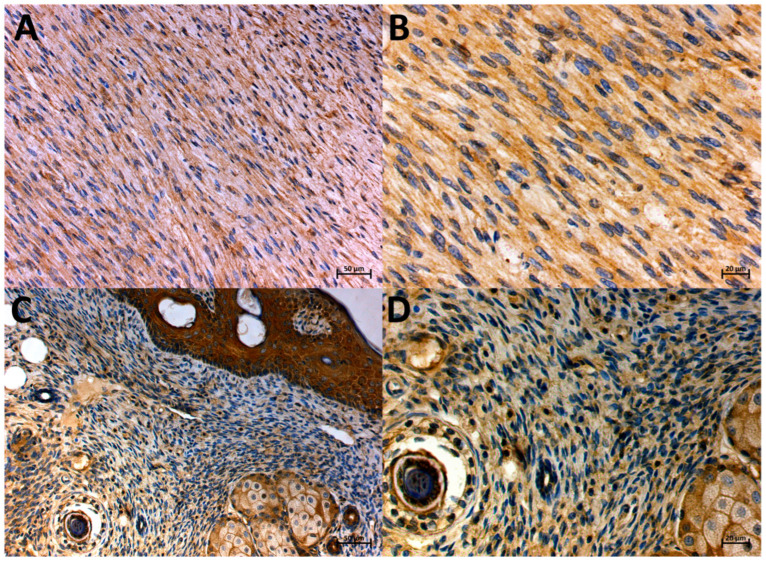
Localization of the MMP-9-expressing cells in FISS (**A**,**B**) and non-FISS (**C**,**D**), immunohistochemically stained. Brown color indicates positive staining. Positive immunostaining for MMP-9 in FISS, intensity 3, 96% of positive cells, magnification 20× and 40×, respectively (**A**,**B**). Positive immunostaining for MMP-9 in non-FISS, intensity 3, 90% of positive cells, magnification 20× and 40×, respectively (**C**,**D**).

MMP- 9 expression was predominantly observed in the cytoplasm of mononucleated spindle cells (Figure 4A,B). Of all the sarcomas (both FISS and non-FISS), 90.2% of the tumors were positive for MMP-9. The mean MMP-9 expression values were 112.1 (SD, ±4.12) and 215.5 (SD, ±86.95) for non-FISS and FISS, respectively. Additionally, positive staining was observed in the peritumoral endothelial cells of small vessels, particularly in capillaries with perivascular lymphoid aggregates. The statistical analysis showed a significantly higher (*p* ≤ 0.05) expression of MMP-9 in FISS than in non-FISS (Figure 2).

### 3.6. Evaluation of PGP Expression

**Figure 5:**
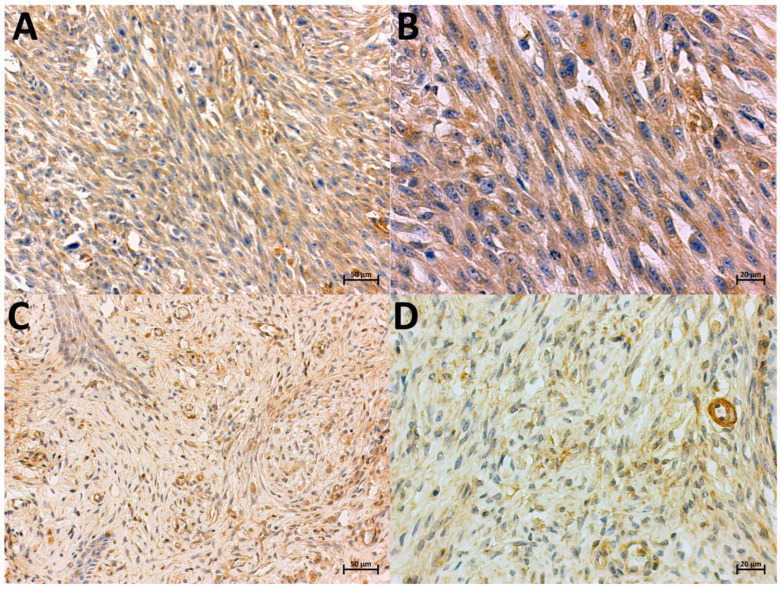
Localization of PGP-expressing cells in FISS (**A**,**B**) and non-FISS (**C**,**D**), immunohistochemically stained. Brown color indicates positive staining. Positive immunostaining for PGP in FISS, intensity 3, 100% of positive cells, magnification 20× and 40×, respectively (**A**,**B**). Positive immunostaining for PGP in non-FISS, intensity 2, 90% of positive cells, magnification 20× and 40×, respectively (**C**,**D**).

PGP expression was predominantly observed in the cytoplasm of the mononucleated spindle cells (Figure 5A,B). The positive expression of PGP was observed in all (100%) evaluated tumors (both FISS and non-FISS). The mean PGP expression values were 4.875 (SD, ±2.748) and 7.909 (SD, ±2.427) for non-FISS and FISS, respectively. The statistical analysis showed a significantly (*p* ≤ 0.05) higher expression of PGP in FISS than in non-FISS (Figure 2).

### 3.7. Correlation between Mitotic Count and PGP, COX-2, MMP-2, and MMP-9 Expressions

**Figure 6:**
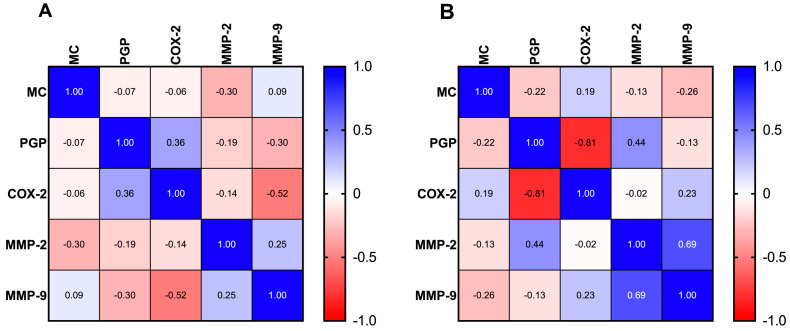
Correlation analysis of the malignancy grade and expressions of P-glycoprotein (PGP), cyclooxygenase-2 (COX-2), matrix metalloproteinase-2 (MMP-2), and matrix metalloproteinase-9 (MMP-9) in feline injection-site sarcoma (FISS) (**A**) and feline noninjection-site sarcoma (non-FISS) (**B**). The correlation was analyzed using a Spearman rank correlation analysis.

A Spearman rank correlation analysis was performed to assess the correlation between the expressions of PGP, COX-2, MMP-2, and MMP-9 and the mitotic count in FISS (Figure 6A) and non-FISS (Figure 6B). A negative correlation between COX-2 expression and MMP-9 expression in FISS (r = −0.52; *p* < 0.01) was observed (Figure 6A). A strong negative correlation between COX-2 expression and PGP expression, and a positive correlation between MMP-2 expression and MMP-9 expression, were observed in non-FISS (r = −0.81; *p* < 0.01) (Figure 6B). There was no statistically significant correlation between the examined proteins and the MC; therefore, it was considered insignificant.

## 4. Discussion

This study aimed to investigate the expression of COX-2, MMP-2, MMP-9, and PGP in FISS and non-FISS, to assess their cross-correlation and statistical differences, so as to expand knowledge about their biological features and possibly employ the findings in further diagnosis.

For that reason, we assessed the expression of the proteins typically involved in inflammatory reactions and related to carcinogenesis. We observed that COX-2, which was shown to have an increased expression in neoplastic tissues in several studies [47,48], was significantly higher in FISS than in non-FISS (*p* ≤ 0.001). This may be related to the fact that local inflammation is an important factor in the formation of FISS, and it is not always observed in non-FISS. Moreover, COX-2 overexpression is associated with several aspects of malignancy, such as the regulation of growth and cell proliferation, an increased ability to evade apoptosis and the immune response, neovascularization, and an increased invasive potential and metastatic dissemination [16,49,50,51]. A strict correlation between chronic inflammatory processes and carcinogenesis has been observed in tumors with a high COX-2 expression [52].

We found a positive expression of COX-2 in 100% of FISS cases and 37.5% of non-FISS cases. Similar results were reported by Magi et al. (2010), who found that COX-2 expression was positive in 97% of FISS cases [53]. In another study by Carneiro et al. (2019), the expression was 61.9% [40]. In contrast, Beam et al. (2003) showed the absence of COX-2 expression in FISS using immunohistochemistry [13]. This discrepancy between results can be associated with using different antibodies and antigen retrieval methods. To the best of our knowledge, there is no study assessing COX-2 expression in non-FISS. 

Further, we demonstrated a moderate negative correlation between COX-2 expression and MMP-9 expression in FISS. There is no study investigating a similar correlation in animal tumors; however, in human tumors such as breast cancer, a positive correlation between COX-2 and MMP-9 expression has been reported, which is the opposite to the results obtained in this study [54]. The possible explanation might be related to the use of anti-inflammatory drugs (NSAIDs) in animals, prior to sample collection. It has been previously described that NSAIDs can modulate COX-2 expression [55,56]. Unfortunately, we are not in possession of the detailed treatment history. Similarly, this finding might be the reason for the strong negative correlation between COX-2 and PGP expression that we obtained in non-FISS, whereas recent studies have demonstrated the opposite correlation in canine transitional cell carcinoma [57,58]. 

COX-2 and its potential role in FISS progression may contribute to a better understanding of the tumor’s behavior, and it may become a target in potential therapy with nonsteroidal anti-inflammatory drugs to treat FISS. However, future investigation is needed.

As previously mentioned, we did not observe a statistically significant difference between MMP-2 expression in non-FISS and in FISS, which is consistent with the results reported by Sorensen et al. (2004) [29]. There is limited information about MMP-2 expression in non-FISS in the available literature. Porcellato et al. (2017) did not consider MMP-2 expression as a useful prognostic marker for FISS [9]. Jankowski et al. (2002) used gel zymography and observed a higher concentration of MMP-2 in sarcomas and carcinomas in comparison to healthy tissues in cats [59]. However, no differences in MMP-2 concentrations between sarcomas and carcinomas were found, which might be related to a different methodology used during the studies. However, gel zymography does not allow a differentiation between cells derived from healthy and from neoplastic tissues. Therefore, the influence of inflammatory components that may contribute to metalloproteinase production cannot be excluded.

Jankowski et al. (2002) [59] showed a high MMP-9 activity in sarcomas and carcinomas in cats. Further, Yasumitsu et al. (1992) [60] demonstrated that MMP-9 had a greater ability (almost 25 times greater) to degrade the extracellular matrix, and is more strongly associated with the destruction of the basement membrane, than MMP-2. Moreover, Yasumitsu et al. (1992) [60] stated that FISS is characterized by a greater malignancy than non-FISS, based on tumor necrosis, mitotic activity, cellular pleomorphism, and the presence of giant multinucleated cells [60]. These results are consistent with those obtained in our study, as MMP-9 expression was significantly higher in FISS than in non-FISS. 

Porcellato et al. (2017) demonstrated a high level of MMP-9 expression in FISS using an immunohistochemical method and concluded that MMP-9 was not a useful prognostic marker for these tumors in cats [9]. However, Porcellato et al. (2017) [9] examined MMP-9 only in FISS. Our study compared the MMP-9 expression in FISS and in non-FISS. We found a positive correlation between MMP-2 and MMP-9 expression in non–FISS, and no correlation in FISS, which is in agreement with Sorensen et al. (2004), who reported no relationship between MMP-2 and MMP-9 expression in FISS [29]. MMP-2 activates latent MMP-9, thus stimulating a specific cascade to produce large amounts of MMP-9 [61], which could explain the correlation reported in our study in non-FISS.

Previous studies have described PGP expression in various types of tumors in cats [34,35]. Brenn et al. (2008) [34] showed a high PGP expression in 54 of 63 feline lymphoma samples. Additionally, a high PGP expression was observed in all feline primary lung cancers (13 adenocarcinomas and 5 squamous cell carcinomas) by Hifumi et al. (2010) [35]. We found PGP expression in all (100%) of the examined tumors (both FISS and non-FISS). In our study, we had no access to the medical history and patient’s treatment, which could be a factor influencing the results obtained by us. For example, it is unclear whether chemotherapy can increase PGP expression in cats. In case of recurrence, lymphoma in cats is often characterized by a greater drug resistance than the one initially observed with primary tumors; hence, it is likely that chemotherapeutics may induce PGP expression in this species [3]. Our results showed a significant difference between PGP expression in FISS and in non-FISS. As there is no available literature comparing the PGP expression in FISS and non-FISS, the results of the present study provide insights about PGP expression in cat’s tumors. Further studies comparing the correlation between PGP expression and previous chemotherapy treatment, as well as tumor malignancy, should be performed to fully assess the role of PGP in FISS and non-FISS.

In the available literature, there is a great variability in the levels of protein expression in FISS and non-FISS. This may be related to the different techniques and methods utilized among the studies. Moreover, different antibodies against various epitopes of proteins have been used in other studies.

In our study, we performed an immunohistochemistry, based on its advantage of providing information about cell distribution and the intensity of enzyme expression; however, its sensitivity is lower than that of Western blot. Western blot provides information about the nature of the isoform that is being recognized, but it does not allow a discrimination between the proteins located in neoplastic cells versus inflammatory and stromal cells around the tumor, which was our area of interest. This way, we could exclude the influence of inflammatory infiltrate around the tumor interfering with protein expression. Nonetheless, to enable a better elucidation of the differences between FISS and non-FISS, further molecular studies are necessary.

## 5. Conclusions

Our results indicated the higher expression of COX-2, PGP, and MMP-9 in FISS in comparison to non-FISS. The evaluated proteins are involved in inflammation and carcinogenesis. Considering the differences in their expression in FISS and non-FISS can contribute to expanding knowledge about their biology and could explain their behavioral diversity. However, further analyses of a higher number of samples, and their correlation with clinical data, are necessary.

## Figures and Tables

**Table 1:** Clinicopathological, histopathological, and immunohistochemical results from utilized samples of FISS and non-FISS (M—male; F—female; MC—mitotic count).

Breed	Gender	Age (Years)	Type of Tumor	Tumor Location	COX-2 Score	MMP-2 Score	MMP-9 Score	PGP Score	MC
Domestic shorthair	F	11	Non-FISS	Gingiva	3	90	140	3	3
Domestic shorthair	M	4.5	Non-FISS	Concha of ear	3	86	200	3	3
Domestic shorthair	F	8	Non-FISS	Abdominal skin (area of mammary gland)	0	70	40	9	3
Domestic shorthair	F	10	Non-FISS	Concha of ear	0	225	270	9	2
Domestic shorthair	F	12	Non-FISS	Abdominal skin (area of mammary gland)	2	45	80	3	3
Domestic shorthair	M	5	Non-FISS	Oral cavity	2	0	60	3	3
Domestic shorthair	M	11	Non-FISS	Facial area	3	40	60	3	2
Domestic shorthair	F	12	Non-FISS	Abdominal skin (area of mammary gland)	0	70	54	6	3
Domestic shorthair	F	4	FISS	Interscapular area	4	0	258	9	3
Domestic shorthair	M	9	FISS	Interscapular area	6	288	297	9	2
Domestic shorthair	M	11	FISS	Interscapular area	8	0	192	6	2
Domestic shorthair	F	8	FISS	Interscapular area	6	0	150	6	3
Domestic shorthair	M	7	FISS	Interscapular area	2	28	288	6	3
Domestic shorthair	F	12	FISS	Interscapular area	8	174	279	9	3
Domestic shorthair	M	4	FISS	Interscapular area	8	170	10	6	2
Domestic shorthair	F	14	FISS	Interscapular area	6	172	146	6	2
Domestic shorthair	F	8	FISS	Interscapular area	6	150	270	2	2
Domestic shorthair	M	15	FISS	Interscapular area	12	80	270	6	3
Domestic shorthair	F	8	FISS	Interscapular area	8	81	210	6	2

**Table 2:** MC results in FISS and non-FISS tumors presented as percentage of all samples [%].

	MC 1	MC 2	MC 3
FISS	0%	57.14%	36.36%
Non-FISS	0%	75.00%	25.00%

## Data Availability

All data generated or analyzed during this study are included in this published article.

## Supplementary Materials

The following supporting information can be downloaded at: https://www.mdpi.com/article/10.3390/ani14142110/s1, Supplementary Material S1. Evaluation of COX-2, MMP-2, MMP-9, and PGP staining in FISS and non-FISS.
