# Supplementary material for: Comparison of MMP-2, MMP-9, COX-2, and PGP Expression in Feline Injection-Site and Feline Noninjection-Site Sarcomas—Pilot Study
Source: Animals (Basel). 2024 Jul 19;14(14):2110. doi: 10.3390/ani14142110 (PMC11273489; doi:10.3390/ani14142110)
Supplement: Supplementary file 1 [file animals-14-02110-s001.zip › animals-3069234-supplementary.pdf]

Supplementary Material S1 Evaluation of COX-2, MMP-2, MMP-9 and PGP staining in FISS and non-FISS.

	**COX-2**		**MMP-2**		**MMP-9**		**PGP**	
**Type of tumor**	**% of positive cells**	**Intensity**	**% of positive cells**	**Intensity**	**% of positive cells**	**Intensity**	**% of positive cells**	**Intensity**
1. Non-FISS	58	1	45	2	70	2	90	1
2. Non-FISS	60	1	43	2	100	2	100	2
3. Non-FISS	6	1	60	1	40	1	100	2
4. Non-FISS	0	0	75	3	90	3	53	3
5. Non-FISS	25	1	45	1	80	1	100	3
6. Non-FISS	20	1	0	0	30	2	80	3
7. Non-FISS	50	1	45	1	30	2	75	3
8. Non-FISS	0	0	35	2	30	2	100	3
9. FISS	90	1	0	0	86	3	80	3
10. FISS	39	2	96	3	96	3	100	3
11. FISS	90	2	0	0	96	2	100	2
12. FISS	60	2	0	0	75	2	85	2
13. FISS	24	1	28	1	96	3	100	2
14. FISS	100	2	87	2	93	3	100	3
15. FISS	70	2	85	2	10	1	100	2
16. FISS	60	2	86	2	73	2	100	3
17. FISS	60	2	50	3	90	3	55	1
18. FISS	80	3	40	2	90	3	100	2
19. FISS	90	2	81	1	70	3	100	2
